# Caregiver´s perception of the home-based management of childhood malaria in Baneghang and Fombap health areas in the West Region of Cameroon

**DOI:** 10.11604/pamj.2022.42.91.28242

**Published:** 2022-06-02

**Authors:** Esther Kenfack Dongmo, Nicholas Tendongfor, Abdel Jelil Njouendou, Dickson Shey Nsagha

**Affiliations:** 1 Department of Public Health and Hygiene, Faculty of Health Sciences, University of Buea, P.O Box 12, Buea, Cameroon,; 2 Department of Biomedical Sciences, Faculty of Health Sciences, University of Buea, P.O Box 12, Buea, Cameroon

**Keywords:** Malaria, perception, knowledge, attitudes, practices, home-based management, home caregivers, Cameroon

## Abstract

**Introduction:**

adequate community perception of malaria is crucial to improving prevention, diagnosis, and treatment. This study aimed to determine the caregiver´s perception of the home-based management of childhood malaria in Baneghang and Fombap health areas, Cameroon.

**Methods:**

a cross-sectional study was carried out to assess the knowledge, attitudes, and practices of home caregivers (HCGs) in Baneghang, a health area under Community Directed Intervention (CDI), and Fombap, a CDI-free health area. Community health workers (CHWs) conducted a census to identify households with under-five children that constituted a sample frame, and then a systematic random sampling was used to select HCGs. Analysis of variance was used to compare the mean score perceptions of HCGs in the management of childhood malaria at the two sites.

**Results:**

out of 420 HCGs interviewed, 161 (38.3%), 226 (53.8%), and 271 (64.5%) displayed correct knowledge, positive attitude, and good practice, respectively, towards childhood malaria prevention, diagnosis, and treatment in both study sites. The mean score knowledge of HCGs in Baneghang was significantly higher than that of those in Fombap (7.33 versus 6.94, p < 0.001). The mean score of attitudes of HCGs towards childhood malaria was also higher in Baneghang than in Fombap (6.59 versus 6.29, p=0.013). However, the mean scores of good practices of HCGs on childhood malaria in both health areas were similar (5.94 versus 5.92, p=0.921).

**Conclusion:**

home-based management of childhood malaria seems to have contributed to good knowledge and positive attitudes of HCGs on malaria prevention, diagnosis, and treatment.

## Introduction

Malaria is one of the leading causes of childhood morbidity and mortality in Africa [1,2]. Each year, 0.7 to 2.7 million people die of malaria, of whom more than 75% are African children [3-5]. In 2017, 435,000 deaths were reported from malaria in the world with most of the cases from Africa, of which the majority were children under five years old, with one child dying every two minutes from this preventable and curable disease [6]. During the same year, like the 10 most affected African countries, Cameroon recorded an increase of about 131,000 additional cases of malaria compared to the previous year [6].

Despite considerable progress in malaria control over the past decade, this infectious disease remains a major public health problem in Cameroon [7,8]. Malaria was responsible for 53% hospitalizations of children under the age of five in Cameroon health facilities in 2017. It was also responsible for 13% of deaths from all causes, and 61% of these deaths concerned children under five years of age [9]. To increase households´ access to healthcare, the national program adopted in 2015 the integrated Community Directed Interventions (CDI) strategy [10-12]. In this program, multi-skilled Community Health Workers (CHWs) were trained to take charge in the community of the main killer disease of children, including malaria, and to strengthen the promotion and prevention of the disease through educational talks and home visits [11]. Home-based management of malaria, including diagnosis by Rapid Diagnosis Test (RDT) and treatment based on test results, is a promising strategy to improve the access of remote populations to prompt and effective management of uncomplicated malaria and to decrease mortality due to malaria [13]. Previous studies showed that Home management of malaria (HMM) reduces the progression of severe malaria by more than 50% and the overall mortality of children under five by 40% [14,15]. Conceptually, HMM involves the health education, prevention, and treatment of malaria in the community by trained CHWs. It is based on the evidence that well-trained and supervised community health workers (CHWs) can provide prompt and adequate treatment of fever cases within 24 hours to help reduce the morbidity and mortality from malaria among under-five children [16].

Implementing this new program has been reported to influence the malaria perception (knowledge, attitudes, and practices) of home caregivers on childhood malaria [17]. Good knowledge and practices in malaria management and prevention in the community can enhance the fight against this disease [18]. Adequate community perception about malaria is crucial in improving prevention, diagnosis, and treatment. According to WHO, the development of effective strategies to impart skills and knowledge to caregivers should be based on an understanding of their current knowledge and behaviour in recognizing and managing malaria [19]. This can be assessed through a situational analysis of the knowledge, prevailing attitudes, and practices relating to malaria in communities [19]. Misconceptions about malaria transmission and its causes still exist. Knowledge about preventive measures does not necessarily translate into an improvement in practices [20]. In a community-based study conducted in Uganda, convulsions, a common complication of malaria, were perceived as a supernatural ailment, best treated by traditional medicine, as was splenomegaly [21]. More than 70% of patients with malaria had treatment from non-public health sources. This included self-treatment, the use of traditional healers, and the use of private medical practitioners or pharmacists [21]. Only 26% used bed nets to prevent malaria, and people who did not use bed nets reported discomfort because of heat and humidity, as well as their high cost [21].

Major barriers to the prevention of malaria in Africa reported in a systematic review included a lack of understanding of its cause and transmission, the belief that it cannot be prevented, and the use of ineffective preventive measures [22]. Another identified specific barrier to the treatment of childhood malaria was the belief that a child with convulsions could die if given an injection or taken to a hospital [22]. In a study conducted in Douala, Cameroon, some participants assimilated the signs and symptoms of malaria to witchcraft, while it was considered by others as an infection of the spleen. They believed taking their affected child to the traditional doctor was a promising solution to remedy the situation [23]. However, the findings of a study in South-West Cameroon revealed that proper education of villagers, particularly mothers, on malaria, and the presence of health facilities where treatment is readily available at an affordable cost close to villages, are important strategies that would significantly reduce malaria morbidity and mortality [24]. For successful implementation of community management of malaria, the proper education to ameliorate the perception (knowledge, attitudes, and practices) of the population needs to be considered. This study aimed to determine the caregiver´s perception of the home-based management of childhood malaria in Baneghang and Fombap health areas. We therefore assessed and compared the level of knowledge, attitude, and practices towards diagnosis, prevention, and treatment of childhood malaria in the two sites.

## Methods

**Study area:** this study was conducted in the localities of the West Region of Cameroon. These included Baneghang health area in Penka-Michel Health District, where integrated CDI was implemented, and Fombap health area in Santchou Health District, which is a non-CDI area.

The Baneghang health area is one of the most populated in the Penka-Michel Health District, with a population of 13,943 inhabitants. It is a rural health area with five health facilities. The number of children under the age of five is estimated to be 2,152 [25]. The main activity of the population is farming and small businesses centered around the commercialization of farm products.

The Fombap health area is the most populated health area in the Santchou Health District, with a population of 6,039 inhabitants and a number of children under five of approximately 1,115 [26]. Its topography is made up of plains and mountains. The hydrology is made up of the main watercourse and their small tributaries providing the area with swampy places that serve as breeding environment for mosquitoes. This health area has 14 villages and is composed mainly of Mbo, Bamileke, and Anglophones [26]. The main activities are agriculture, petty trading, and breeding. The Fombap health area has four health facilities [26].

**Study design and population:** a cross-sectional study was carried out in the Baneghang and Fombap health areas to evaluate the home caregivers' perception of the home-based management of childhood malaria. The Community Directed Intervention (CDI) for malaria has been previously implemented in the Baneghang health area while Fombap still remains CDI-free. The level of knowledge, attitudes, and practices (KAP) of home caregivers towards prevention, diagnosis, and treatment of childhood malaria were evaluated in both sites. The target population was HCGs who take care of children under five in both study health areas.

**Selection criteria:** households with at least one child under five years old and their home caregivers residing in the Baneghang and Fombap health areas were included in this study. Home caregivers included in the study were aged 18 to 60 years.

**Sample size determination:** the minimum sample size required for this cross-sectional study for a 50% proportion of outcome, 95% confidence level, and 5% margin of error was 384. However, as we intended to compare the mean scores of knowledge, attitude and practices of both study sites, given the limited number of households in the Fombap health area, as compared to Baneghang, using a ratio of 1: 2, assuming an effect size of 0.3, the minimum number of HCGs expected to achieve a statistical power of 80% was 262 in Baneghang and 131 in Fombap [27]. We finally recruited 277 and 143 HCGs at both sites, respectively.

**Sampling methods:** within each community in Baneghang and Fombap health areas, CHWs conducted a census to identify households systematically with under-five children that constituted a sampling frame. In the last stage, a systematic random sample of 420 households with the corresponding number of home caregivers in the two areas were selected and enrolled for the study; thus, 277 home caregivers in the Baneghang health area and 143 in the Fombap health area. The sampling interval for each community was calculated by dividing the total number of households with under-five children by the sample size a priori (N / n). Thus, each household was selected in the sampling interval of 1-2.

**Data collection tool:** the questionnaire for this study was adapted from the survey on the knowledge, attitudes, and practices of malaria in Togo [28]. This questionnaire captured socio-demographic and family characteristics, home caregivers´ malaria attitudes, home caregivers´ malaria practices, and home caregivers´ malaria behaviours. The questionnaire was pretested in the Nkongso health area in the Mifi Health District of the West Region of Cameroon, adjacent to Baneghang health area, with its population sharing similar socio-demographic characteristics.

**Data collection procedures:** a structured questionnaire was administered to selected home caregivers in the Baneghang and Fombap health areas to capture related data. i) Before data collection, a field trip was organized to contact the CHWs´ resource persons in each community. They were facilitated not only by identifying households where there were under-five children, but also by facilitating access to these households. ii) Data collectors were trained and supervised to interview home caregivers of children under five in their household. iii) The interviews were conducted at their convenient times of the day, and each session was estimated to last about 45 minutes. Caregivers from selected households were invited to respond to the questionnaires by trained data collectors, since most of the respondents were not able to read and write. Data collectors read each question as written (verbatim), including all possible responses, and recorded the participants´ responses. Where feasible, the resource person preferably translated the questions into the “local language”. In the event that participants do not understand or give an ambiguous response, the data collector probed for a specific response from the respondents.

**Scoring the level of knowledge, attitudes, and practices:** for analysis of the knowledge section, a total of 8 items were included: elementary knowledge of malaria, its signs and symptoms, complications, awareness of the free treatment of uncomplicated malaria in under-five children, and prevention. For the eight items of knowledge questions, the maximum attainable score was “8” and the minimum score was “0”. Likewise, in the attitudes section, a total of 8 items were included, which consisted of respondents' attitudes towards childhood malaria. A Likert scale was used to measure attitudes. Questions evaluating attitudes towards home-based prevention, diagnosis, and management of childhood malaria were asked. Each positive response (agree) was assigned a score of “1”, and each negative response a score of ‘0´. For the eight attitude-related questions, the maximum attainable score was “8” and the minimum score was “0”. From the same perspective, for the nine items in the practice category, such as consulting a nurse or CHW, taking temperature, doing an RDT, taking prescribed ACTs, and using Long-lasting insecticidal nets (LLINs), the maximum attainable score was ‘9´ and the minimum was ‘0´.

**Data management and analysis:** all data were checked for accuracy, completeness, and consistency at the end of each day by one of the supervisors. Data was entered into a form designed using Epi-data version 3.1. An analysis of variance was used to compare the mean scores of the knowledge, attitudes, and practices of home caregivers in the management of childhood malaria in the Baneghang and Fombap health areas. The chi-square test and Odds ratio were used to measure the association between the level of perception and the study area. All p-values of less than 0.05 were considered statistically significant.

**Administrative and ethical approvals:** the principles of good ethical research involving human participants were respected. The protocol of the study was approved by the Institutional Review Board of the Faculty of Health Sciences of the University of Buea (IRB-FHS No: 2019/1023-09/UB/SG/IRB/FHS). Administrative authorization was obtained from the Regional Delegate of Public Health of the West Region of Cameroon and the District Medical Officers of Penka-Michel and Santchou.

## Results

**Socio-demographic characteristics of home caregivers:** Table 1 shows the sociodemographic characteristics of the home caregivers (HCGs) in the Baneghang health area (under integrated CDI) and Fombap health area (non-CDI). A total of 420 home caregivers (HCGs) were interviewed; 277 (66.0%) in Baneghang, and 143 (34.0%) in Fombap. Both sites were homogenous in terms of gender, education, occupation, wealth perception, and the number of under-five children. However, the distribution of participants´ age and religion differed significantly between the two sites.

**Table 1:** socio-demographic profile of HCGs in the Baneghang and Fombap health areas, 2019

Variables	Baneghang HA N0 (%)	Fombap HA N0 (%)	Total N0 (%)	Chi-square value	p-value
**Age (years)**				**12.555**	**0.002**
< 21	21 (7.6)	14 (9.8)	35 (8.3)		
21-40	127 (45.8)	88 (61.5)	215 (51.2)		
41+	129 (46.6)	41 (28.7)	170 (40.5)		
Total	277 (66.0)	143 (34.0)	420 (100.0)		
**Gender**				0.013	0.909
Male	34 (12.3)	17 (11.9)	51 (12.1)		
Female	243 (87.7)	126 (88.1)	369 (87.9)		
Total	277 (66.0)	143 (34.0)	420 (100.0)		
**Education**				3.214	0.091
No formal education	22 (7.9)	4 (2.8)	26 (6.2)		
Primary	124 (44.8)	57 (39.9)	181 (43.1)		
Secondary	124 (44.8)	78 (54.5)	202 (48.1)		
Tertiary	7 (2.5)	4 (2.8)	11 (2.6)		
Total	277 (66.0)	143 (34.0)	420 (100.0)		
**Religion**				13.454	0.001
Traditionalist	171 (61.7)	116 (81.1)	287 (68.3)		
Muslim	2 (0.7)	0 (0.0)	2 (0.5)		
Christian	104 (37.5)	27 (18.9)	131 (31.2)		
Total	277 (66.0)	143 (34.0)	420 (100.0)		
**Occupation**				1.004	0.263
Housewife	81 (29.2)	54 (37.8)	135 (32.1)		
Student	11 (4.0)	10 (7.0)	22 (5.0)		
Farmer	141 (50.9)	57 (39.9)	198 (47.1)		
Employed	81 (29.2)	54 (37.8)	135 (32.1)		
Total	277 (66.0)	143 (34.0)	420 (100.0)		
**Number of children < 5yrs**				1.024	0.374
1	128 (46.)	56 (39.2)	184 (43.8)		
2	100 (36.1)	57 (39.9)	157 (37.4)		
3+	49 (17.7)	30 (21.0)	79 (18.8)		
Total	277 (66.0)	143 (34.0)	420 (100.0)		
**Wealth perception**				2.547	0.064
Very poor	11 (4.0)	12 (8.4)	23 (5.5)		
Poor	162 (58.5)	66 (46.2)	228 (54.4)		
Rich	9 (3.2)	5 (3.5)	14 (3.3)		
Averagely rich	93 (33.6)	60 (42.0)	153 (36.4)		
Very rich	2 (0.7)	0 (0.0)	2 (0.5)		
Total	277 (66.0)	143 (34.0)	420 (100.0)		

**Children´s malaria: correct knowledge, positive attitudes, and good practices for home caregivers:** out of 420 HCGs interviewed, 161 (38.3%), 226 (53.8%), and 271 (64.5%) respectively displayed correct knowledge, positive attitudes, and good practices towards childhood malaria in both study sites. When compared to their counterparts in the non-CDI site, a high proportion of HCGs in the Baneghang health area had significantly correct knowledge (46.2 versus 23.1, p = 0.001), positive attitudes (58.1 versus 45.5, p = 0.014), and good practices (68.2 versus 57.3, p = 0.027) on childhood malaria (Table 2). The mean score knowledge of HCGs in the Baneghang health area was significantly higher than that in the Fombap health area (7.33 versus 6.94; p=0.001). The same observations were made with the mean score of attitudes in Baneghang and Fombap health areas (6.59 versus 6.29; p=0.014). However, there was no statistically significant difference in mean score practices between the two sites (5.94 versus 5.92, p=0.921; Table 3).

**Table 2:** children’s malaria, correct knowledge, positive attitudes, and good practices for home caregivers, 2019

Perception	Item	Baneghang No (%)		Fombap No (%)		Total No (%)	P-value
Knowledge		Correct	Incorrect	Correct	Incorrect		
	Ever heard of malaria	268 (97.5)	9 (2.5)	137 (95.8)	6 (4.2)	405 (96.9)	0.354
	Knows fever is the cardinal sign of malaria	272 (98.2)	5 (1.8)	137 (95.8)	6 (4.2)	409 (97.4)	0.147
	Knows malaria is transmitted through mosquito bites	271 (97.8)	6 (2.2)	136 (95.1)	7 (4.9)	407 (96.9)	0.126
	Knows malaria is prevented by cleaning surroundings and using mosquito nets	276 (99.6)	1 (0.4)	140 (97.9)	3 (2.1)	416 (99.0)	0.087
	Know childhood malaria can be confirmed by checking body temperature and using an RDT	268 (96.8)	9 (3.2)	141 (98.6)	2 (1.4)	409 (97.4)	0.265
	Knows malaria is treated either in the hospital or CHW	249 (89.9)	28 (10.1)	118 (82.5)	25 (17.5)	367 (87.4)	0.031*
	Knows the treatment of uncomplicated malaria in children 0-5 years is free	152 (54.9)	125 (45.1)	43 (30.1)	100 (69.9)	195 (46.4)	0.001*
	Knows anemia and even death are the consequences of poorly treated malaria in children	274 (98.9)	3 (1.1)	141 (98.6)	2 (1.4)	415 (98.8)	0.778
	**Overall knowledge**	**128 (46.2)**	**149 (53.8)**	**33 (23.1)**	**110 (76.9)**	**161 (38.3)**	0.001*
**Attitudes**		**Positive**	**Negative**	**Positive**	**Negative**		
	Agrees children run a risk of getting malaria	254 (91.7)	23 (8.3)	131 (91.6)	12 (8.4)	385 (91.7)	0.975
	Thinks mosquito nets can prevent malaria	247 (89.2)	30 (10.8)	120 (83.9)	23 (16.1)	367 (87.4)	0.124
	Considers mosquito nets to be good	209 (75.5)	68 (24.5)	112 (78.3)	31 (21.7)	321 (76.4)	0.512
	Perceives malaria as a serious disease	250 (90.3)	27 (9.7)	134 (93.7)	9 (6.3)	384 (91.4)	0.231
	Thinks malaria is easily preventable	167 (60.3)	110 (39.7)	68 (47.6)	75 (52.4)	235 (56.0)	0.013*
	Agrees that one must be tested before giving any anti-malaria	219 (79.1)	58 (20.9)	106 (74.1)	37 (25.9)	325 (77.4)	0.252
	Thinks eating lots of mangoes and unripe fruits can give malaria	230 (83.0)	47 (17.0)	112 (78.3)	31 (21.7)	342 (81.4)	0.239
	Thinks malaria can be gotten by staying under the rain	250 (90.3)	27 (9.7)	116 (81.1)	27 (18.9)	366 (87.1)	0.008*
	**Overall attitudes**	**161 (58.1)**	**116 (41.9)**	**65 (45.5)**	**78 (54.5)**	**226 (53.8)**	0.014*
**Practices**		**Good**	**Poor**	**Good**	**Poor**		
	Takes child to a CHW or HF when malaria or fever is suspected in the child	239 (86.6)	38 (13.4)	101 (70.6)	42 (29.4)	340 (81.1)	0.001*
	Consulted a CHW/Nurse when the child had fever	190 (68.6)	87 (31.4)	85 (59.4)	58 (40.6)	275 (65.6)	0.004*
	Blood test was done to confirm fever	197 (71.1)	80 (28.9)	79 (55.2)	64 (44.8)	276 (65.7)	0.001*
	Tested positive for malaria	161 (58.1)	116 (41.9)	69 (48.3)	74 (51.7)	230 (54.8)	0.002*
	Anti-malarial was prescribed	188 (94.9)	89 (5.1)	80 (98.8)	63 (1.2)	268 (96.1)	0.137
	Bought anti-malarial	152 (82.2)	125 (17.8)	76 (93.8)	67 (6..2)	228 (85.7)	0.012*
	Owns a LLINs	262 (96.0)	15 (4.0)	143 (100)	00 (00)	405 (97.4)	0.015*
	Child sleeps systematically under LLINs	243 (88.4)	34 (11.6)	139 (97.2)	4 (2.8)	382 (91.4)	0.002*
	Wears long sleeves and use insecticides to prevent mosquito bite	197 (71.4)	80 (28.6)	115 (81.0)	28 (19.0)	312 (74.6)	0.032*
	**Overall practices**	**189 (68.2)**	**88 (31.8)**	**82 (57.3)**	**61 (42.7)**	**271 (64.5)**	0.027*

* = Significant

**Table 3:** summary statistics of HCG knowledge attitudes and practices scores in the management of childhood malaria in the Baneghang and Fombap health areas, 2019

Summary statistics						
Perception	Health area	Sample size	Total score	Mean score	Variance	Standard deviation
**Knowledge**	Fombap	143	992	6.94	0.62	0.79
	Baneghang	277	2030	7.33	0.55	0.74
**Attitudes**	Fombap	143	899	6.29	1.36	1.17
	Baneghang	277	1825	6.59	1.44	1.20
**Practices**	Fombap HA	143	847	5.92	4.45	2.11
	Baneghang HA	277	1645	5.94	5.38	2.32
**ANOVA**						
**Perception**	**Variation**	**Sum of square**	**Degree of freedom**	**Mean square**	**F-statistic**	**p-value**
**Knowledge**	Between groups	13.940	1	13.940		
	Within groups	238.657	418	0.571	24.416	< 0.001
	Total	252.598	419			
**Attitudes**	Between groups	8.793	1	8.793		
	Within groups	590.147	418	1.412	6.228	0.013
	Total	598.940	419			
**Practices**	Between groups	0.048	1	0.048		
	Within groups	2118.950	418	5.068	0.009	0.923
	Total	2118.998	419			

**Association between knowledge, attitudes and practices of HCGs on malaria and the two health areas:** when compared to those in the Fombap health area, HCGs in the Baneghang health area were 2.8 times (OR: 2.8; 95%CI [1.4 - 4.6]; p=0.001) more likely to have the correct knowledge, 1.7 times (OR: 1.7; 95%CI [1.1 - 2.7]; p=0.014) more likely to have positive attitudes, and 1.6 times (OR: 1.6; 95%CI [1.0 - 2.8]; p=0.027) more likely to have good practices with respect to diagnosis, prevention, and management of childhood malaria (Table 4).

**Table 4:** relationship between HCGs´ malaria knowledge, attitudes, and practices and the two health areas, 2019

Perception	Health area	Correct knowledge/Positive attitudes/Good practices No (%)	Incorrect knowledge/Negative attitudes/Poor practices No (%)	OR (95%CI)	p-value
**Knowledge**	Fombap	33 (23.1)	110 (76.9)	1	
	Baneghang (CDI)	128 (46.2)	149 (53.8)	2.8 (1.4-4.6)	0.001*
	Total	161 (38.3)	259 (61.7)		
**Attitudes**	Fombap	65 (45.5)	78 (54.6)	1	
	Baneghang (CDI)	161 (58.1)	116 (41.9)	1.7 (1.1-2.7)	0.014*
	Total	226 (53.8)	194 (41.9)		
**Practices**	Fombap HA	82 (57.3)	61 (42.7)	1	
	Baneghang (CDI)	189 (68.2)	88 (31.8)	1.6 (1.0-2.8)	0.027*
	Total	271 (64.5)	149 (35.5)		

## Discussion

This study investigated the impact of home-based management of malaria on home caregivers´ perceptions of malaria diagnosis, prevention, and treatment in Baneghang and Fombap health areas. These two health areas have different approaches to the management of childhood malaria in their communities. In fact, Baneghang health area is found in the Penka-Michel Health District, where integrated CDI for childhood malaria has been previously implemented for the past four years. The community-directed intervention (CDI) strategy is an approach in which communities themselves direct the planning and implementation of intervention delivery [29]. In the harmonized CDI strategy currently being implemented in Cameroon, the multitasks Community Health Worker should offer a package of services on malaria, acute respiratory infections, diarrhea, tuberculosis, HIV/AIDS, malnutrition, onchocerciasis, diseases preventable by vaccination [30]. The Baneghang health area is one of the most populated of Penka-Michel Health District with a population of 13943 inhabitants. It is a rural health area with 5 health facilities. Children under five are estimated at 2152 [25].

The Fombap health area is located in the Santchou Health District. This health district is a district where the integrated CDI strategy is not implemented. It is one of the districts in the West Region where malaria proportional morbidity is high (44.7%) [31]. The Fombap health area is the most populated rural health area in the Santchou district, with a population of 6,039 inhabitants. Children under five represent about 1,115 people. Its landscape is made up of plains and mountains. There is the main river and its small tributaries that make the area swampy, providing a breeding ground for [26].

Knowledge, attitudes, and practices were assessed as indicators of home caregivers' perceptions about malaria diagnosis, prevention, and treatment in children under five within the study sites. The overall correct knowledge of home caregivers in the Baneghang health area, where the integrated CDI has been implemented for the past four years, was significantly higher compared to the Fombap health area. The improvement in HCGs' knowledge of childhood malaria management is probably due to the effect of integrated CDI, where CHWs make regular home visits to sensitize HCGs on malaria prevention. In this area, home caregivers received health education about malaria prevention measures like cleaning around the house, the use of mosquito nets, window screens, insecticides compared to the Fombap health area where the HCGs did not receive any health education. This result concurs with that of Tobin-West and Briggs were in the experimental group, adequate knowledge of caregivers significantly increased after malaria intervention (trained on malaria and provided with bed nets and artemisinin-lumefantrine to treat children under five who developed a fever during the period of the study) compared to the non-CDI group [32]. There was also a remarkable increase in their knowledge of prevention and case management of malaria, thus correlating the results from other studies where educational interventions on malaria by community volunteers improved knowledge, attitude, and treatment-seeking behavior among caregivers [33].

The overall positive attitude of home caregivers in the Baneghang health area, where the integrated CDI was implemented, was significantly higher compared to the Fombap health area. The more positive attitude of HCGs towards childhood malaria in the Baneghang health area is probably due to all the actions carried out by the CHWs with HCGs in the integrated CDI strategy that could improve their attitudes. This corroborates with the results from Ahmadi *et al*. where the effects of educational intervention on long-lasting insecticidal improved significantly the attitudes of caregivers [34].

The overall good practice of home caregivers in the Baneghang health area in the prevention and management of childhood malaria, where the integrated CDI was implemented, was significantly higher compared to the Fombap health area. This result agreed with the findings of Amoran and Olorunfemi and others where an intervention program, increased the proportion of home caregivers with good practices for malaria prevention [35-37].

## Conclusion

The implementation of home-based malaria management in the Baneghang health area may have contributed to home caregivers´ improved perceptions of malaria diagnosis, prevention, and treatment. Extension of the integrated CDI strategy to areas where it has not yet been implemented would improve caregivers´ knowledge, attitudes, and practices towards the management of malaria and therefore contribute to the reduction of malaria control burden in children through appropriate community interventions.

### What is known about this topic

- Adequate community perception of malaria is crucial to improving prevention, diagnosis, and treatment.

### What this study adds

- knowledge, attitudes, and practices of home caregivers on malaria were significantly higher in the health area where the integrated CDI was implemented compared to the health area where it was not implemented.
